# Relationship between out-of-hospital cardiac arrests and COVID-19 during the first and second pandemic wave. The importance of monitoring COVID-19 incidence

**DOI:** 10.1371/journal.pone.0260275

**Published:** 2021-11-19

**Authors:** Enrico Baldi, Roberto Primi, Sara Bendotti, Alessia Currao, Sara Compagnoni, Francesca Romana Gentile, Giuseppe Maria Sechi, Claudio Mare, Alessandra Palo, Enrico Contri, Vincenza Ronchi, Giuseppe Bergamini, Francesca Reali, Pierpaolo Parogni, Fabio Facchin, Ugo Rizzi, Daniele Bussi, Simone Ruggeri, Sabina Campi, Paola Centineo, Roberto De Ponti, Luigi Oltrona Visconti, Simone Savastano, all the Lombardia CARe researchers

**Affiliations:** 1 Department of Molecular Medicine, Section of Cardiology, University of Pavia, Pavia, Italy; 2 Cardiac Intensive Care Unit, Arrhythmia and Electrophysiology and Experimental Cardiology, Fondazione IRCCS Policlinico San Matteo, Pavia, Italy; 3 Division of Cardiology, Fondazione IRCCS Policlinico San Matteo, Pavia, Italy; 4 Agenzia Regionale Emergenza Urgenza, Milan, Italy; 5 AAT Pavia - Agenzia Regionale Emergenza Urgenza (AREU) c/o Fondazione IRCCS Policlinico San Matteo, Pavia, Italy; 6 ASST di Pavia, Pavia, Italy; 7 AAT Lodi - Agenzia Regionale Emergenza Urgenza (AREU) c/o ASST di Lodi, Lodi, Italy; 8 AAT Mantova - Agenzia Regionale Emergenza Urgenza (AREU) c/o ASST di Mantua, Mantua, Italy; 9 AAT Cremona - Agenzia Regionale Emergenza Urgenza (AREU) c/o ASST di Cremona, Cremona, Italy; 10 AAT Varese - Agenzia Regionale Emergenza Urgenza (AREU) c/o ASST dei Sette Laghi, Varese, Italy; 11 ASST-Settelaghi, Ospedale di Circolo - Università dell’Insubria, Varese, Italy; St. Michael’s Hospital, CANADA

## Abstract

**Background:**

The relationship between COVID-19 and out-of-hospital cardiac arrests (OHCAs) has been shown during different phases of the first pandemic wave, but little is known about how to predict where cardiac arrests will increase in case of a third peak.

**Aim:**

To seek for a correlation between the OHCAs and COVID-19 daily incidence both during the two pandemic waves at a provincial level.

**Methods:**

We considered all the OHCAs occurred in the provinces of Pavia, Lodi, Cremona, Mantua and Varese, in Lombardy Region (Italy), from 21/02/2020 to 31/12/2020. We divided the study period into period 1, the first 157 days after the outbreak and including the first pandemic wave and period 2, the second 158 days including the second pandemic wave. We calculated the cumulative and daily incidence of OHCA and COVID-19 for the whole territory and for each province for both periods.

**Results:**

A significant correlation between the daily incidence of COVID-19 and the daily incidence of OHCAs was observed both during the first and the second pandemic period in the whole territory (R = 0.4, p<0.001 for period 1 and 2) and only in those provinces with higher COVID-19 cumulative incidence (period 1: Cremona R = 0.3, p = 0.001; Lodi R = 0.4, p<0.001; Pavia R = 0.3; p = 0.01; period 2: Varese R = 0.4, p<0.001).

**Conclusions:**

Our results suggest that strictly monitoring the pandemic trend may help in predict which territories will be more likely to experience an OHCAs’ increase. That may also serve as a guide to re-allocate properly health resources in case of further pandemic waves.

## Introduction

An increase in out-of-hospital cardiac arrest (OHCA) incidence has been observed during the first peak of the Coronavirus Disease-19 (COVID-19) pandemic, especially in the most affected countries [1–3]. This OHCAs’ increase has been explained both by infection-related causes and by pandemic-related causes. The most common infection-related causes are the rapid progression of respiratory failure [4], the pulmonary embolism [5], the myocarditis [6], the myocardial injury [7], the myocardial infarction due to a pro-thrombotic state [8] and the arrhythmias due to the SARS-CoV-2 infection and/or as side effect of drugs [9]. On the other hand, fear of in-hospital infection who prevented patients to call the Emergency Medical System (EMS) in case of effective need [10, 11] and the reduction in outpatient clinic visits [12, 13] represented the most common pandemic-related causes, leading to at home deterioration of time-dependent diseases. However, the increase in OHCA incidence has not been observed in all the countries [14], probably as a result of different pandemic burden. Moreover, albeit a correlation between OHCA incidence and COVID-19 case has been suggested both during the ascendant and the descendent phase of the first pandemic wave in the most affected countries [15], it is unknown whether this relationship could be present also during the second pandemic wave. The aim of our study was to analyse the trends of OHCA incidence and COVID-19 along the whole 2020 and to test the presence of a correlation between them during both the first and second pandemic wave at a provincial level.

## Methods

### Study population

The Lombardia Cardiac Arrest Registry (Lombardia CARe: NCT03197142) is a multicentre longitudinal prospective registry, which started enrolling all the OHCAs from the Province of Pavia from January 2015, and then was extended also to the Provinces of Lodi, Cremona, and Mantua from January 2019 and to the province of Varese since January 2020. All the data are collected according to the 2014 Utstein recommendations [16]. It was approved by the Ethical Committee of the Fondazione IRCSS Policlinico San Matteo (proc.20140028219), where the registry is hosted, and by all the others involved in the territory. According to the Ethics approval, the informed consent was required and signed only by patients who survived up to hospital discharge with a good neurologic outcome. The informed consent was waived for all the other cases and the data were anonymised.

For the present study, we considered all the OHCAs occurred in the provinces of Pavia, Lodi, Cremona, Mantua and Varese, in the Lombardy Region, in the northern Italy, after the first documented COVID-19 case in the Region (21^st^ February 2020) and up to the 31^st^ December 2020. The daily new cases and the cumulative incidence of COVID-19, based on the confirmed cases announced by the National Department of Civil Defence/Protezione Civile Nazionale [17], were also computed.

### Setting and Emergency Medical System (EMS) description

The total area covered by the Lombardia CARe registry is of 9,061‬ km^2^ divided into the five provinces (Pavia 2,969 km^2^; Lodi 783 km^2^; Cremona 1,770 km^2^; Mantua 2,341 km^2^; Varese 1,198 km^2^). Each province has several rural regions and a few urban areas for a total population of 2,415,491 (Pavia 540,376; Lodi 227,412; Cremona 355,908; Mantua 406,919; Varese 884,876) as of January 1^st^, 2020.

In the entire Lombardy region, the emergency medical services is provided and coordinated by the “Agenzia Regionale Emergenza Urgenza (AREU)” which ensures the same procedures in all the region and collect all the electronic data from every rescue in a single data warehouse connected by the Lombardia CARe database.

According to the organization of AREU the rescue calls in the study territory are handled by two EMS dispatch centres: the “Sala Operativa Regionale Emergenza Urgenza (SOREU) della Pianura” based in Pavia and covering the provinces of Pavia, Lodi, Cremona and Mantua, and the “SOREU dei Laghi” based in Como and covering the provinces of Varese, Como and Lecco (the last two not already considered in the Lombardia CARe). The dispatch centres coordinates ambulances staffed with basic life support defibrillation (BLS-D)-trained personnel, and advanced life support (ALS)-trained staffed vehicles (a physician and a specialized nurse or a specialized nurse only). Moreover, five helicopters with a physician and a specialized nurse on board also serve the entire Lombardy region and other three can intervene from other neighboring regions. In case of suspected OHCA, the dispatcher activates one to three emergency vehicles (which may include a helicopter) with at least one physician and assists the calling bystander during chest compressions (telephone CPR). The decision about the attempt and the duration of resuscitation are left to the physician, whilst BLS-D-trained personnel are instructed to start resuscitation unless clear signs of death are present (rigor mortis, hypostasis, and injuries not compatible with life).

### Data management

The data of the study are collected and managed using Research Electronic Data Capture (REDCap), a secure, web-based software platform, hosted at Fondazione IRCCS Policlinico San Matteo [18, 19].

### Statistical analysis

We present categorical variables as counts and percentage. We present continuous variables using the median and 25th–75th interquartile range (IQR). Chi-squared test was use for the comparison of categorical variables. Kruskal-Wallis test was used to compared skewed continues variables in the different provinces.

We divided the study period in two equal portions: period 1 namely the first 157 days after the outbreak and including the first pandemic wave (from 21/02/2020 to 26/07/2020) and period 2 covering the second 158 days and including the second pandemic wave (from 27/07/2020 to 31/12/2020). We performed this choice considering both that an official date for both the end of the first pandemic wave and the start of the second one was not present and the following calculation of sample size. Basing on a previous paper from our group focused only on the first wave [15], where a correlation between the daily OHCA and COVID incidence with a coefficient of 0.4 was observed, assuming a possible decrease of the correlation strength due to the increase of the time frame (from 283 days in that paper to 313 in the present one), we calculated a sample size (N) required to determine a statistical significant correlation with a coefficient of at least 0.25. So, considering a type I (α) error of 0.05 and a type II (β) error of 0.2 and applying the following formula [standard normal deviate for α = Zα = 1.9600; standard normal deviate for β = Zβ = 0.8416; r = 0.25; C = 0.5 * ln[(1+r)/(1-r)] = 0.2554; N = [(Zα+Zβ)/C]2 + 3 = 123], the resulting minimum sample size was 123 days.

We calculated the cumulative, the daily and the 7-days incidence of OHCA expressed per 100,000 inhabitants both for the whole territory and for each province separately for both periods. We also calculated the cumulative, the daily and the 7-days incidence of COVID-19 cases per 100,000 inhabitants in the whole territory and in each province separately for both periods. We categorized the different Provinces at high-incidence or at low-incidence of COVID-19 cases depending on whether their cumulative incidences of COVID-19 cases per 100,000 inhabitants were over or beneath the cumulative incidence of the whole territory for each period. Via polynomial regression models we computed the Spearman R and its 95% confidence interval (CI) to measure the strength of the correlation between the daily incidence per 100,000 inhabitants of COVID-19 cases and the daily incidence per 100,000 inhabitants of OHCAs both for the entire series and for each province. Statistical analyses were performed with the MedCalc version 19.2 (MedCalc Software Ltd) and R version 4.0.4 (R foundation). All tests were two-sided, and a P-value<0.05 was considered statistically significant.

## Results

### COVID-19 cumulative incidence

**Fig 1:**
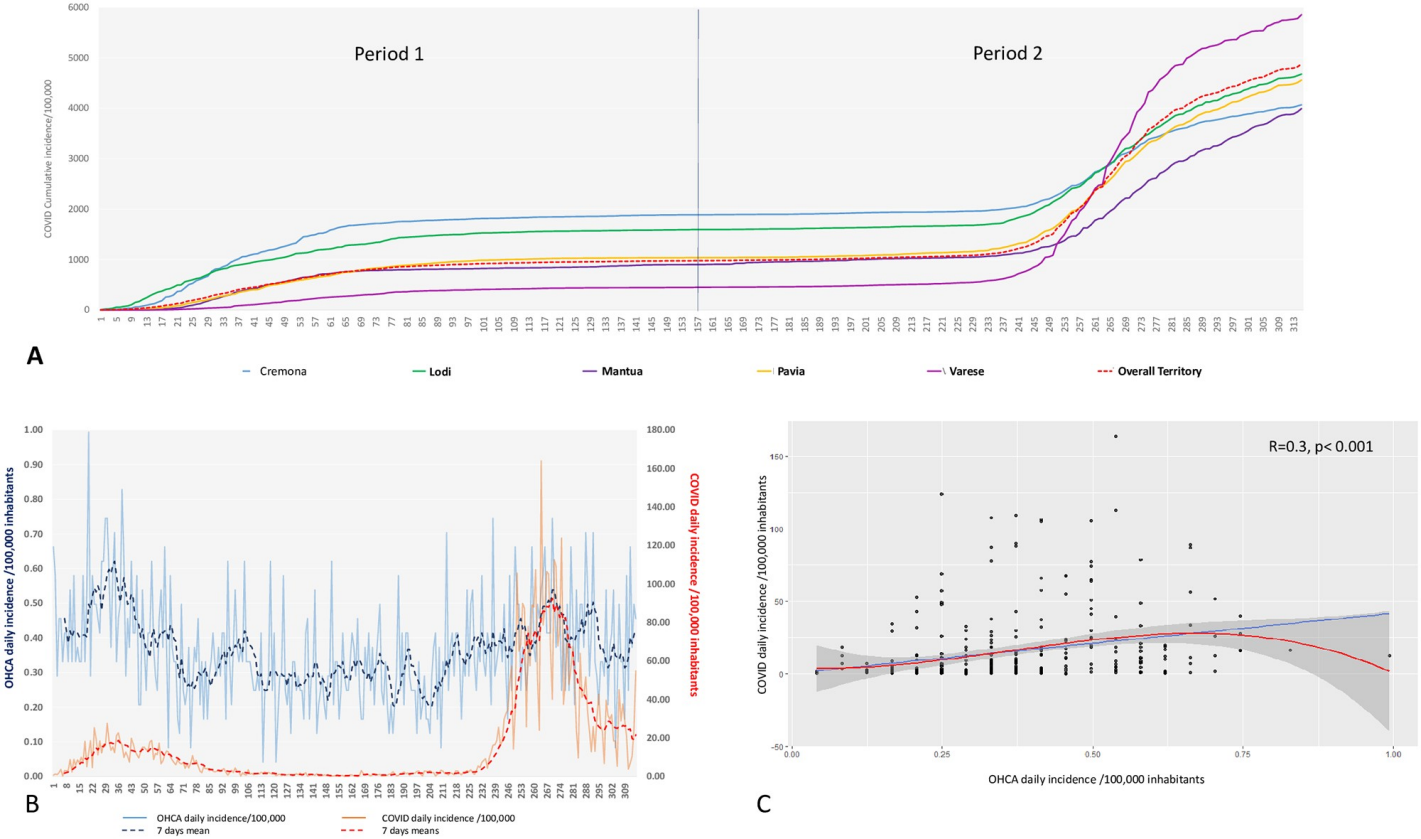
**Panel A)** COVID-19 cumulative incidence per 100,000 inhabitants in the whole territory and in each province separately in the period 1 and in period 2. **Panel B)** Trend of daily (blue continuous line) and 7-days (blue dotted line) OHCA incidence per 100,000 inhabitants and of daily (red continuous line) and 7-days (red dotted line) COVID-19 incidence per 100,000 inhabitants for the whole territory. **Panel C)** Relationship between COVID-19 daily incidence per 100,000 inhabitants (vertical axis) and OHCA daily incidence per 100,000 inhabitants (horizontal axis). The blue line indicates the linear regression and the red line indicates the polynomial regression. The grey area indicates the 95%CI.

The cumulative incidence of COVID-19 in the overall territory was 977.2/100,000 at the end of period 1 and 4875.8/100,000 at the end of period 2 (Fig 1A). Three provinces showed a high COVID-19 incidence in period 1 as they overcame the cumulative incidence of the whole territory: Cremona (1887.3/100,000), Lodi (1588.3/100,000) and Pavia (1038/100,000). On the contrary two provinces were at low COVID-19 incidence: Mantua (902.4/100,000) and Varese (449/100,000). As far as period 2 is concerned, only the province of Varese was at high COVID-19 incidence with 5401.8 case/100,000, overcoming the whole territory, whilst the other four provinces were at low incidence: Pavia (3521/100,000); Mantua (3089.3/100,000); Lodi (3082/100,000) and Cremona (2179/100,000).

### Patients’ characteristics among provinces and periods

The patients’ and OHCAs’ characteristics in the overall population as well as the comparison among the different provinces are presented in Table 1. Sex, EMS arrival time, the OHCA etiology, the OHCA location, the witnessed status, the rate of advanced resuscitation initiated, and the duration of resuscitation were slightly but significantly different amid provinces. On the contrary, the presenting rhythm, the rate of patients with resuscitation attempted and the rate of ROSC were similar. Concerning the comparison between patients’ and OHCAs’ characteristics in period 1 vs period 2, the home location of OHCA was higher in period 1, whilst the rate of both dispatcher-assisted CPR and of advanced resuscitation were higher during period 2 (Table 2). Comparing the provinces at high vs low COVID-19 incidence in the two periods, no differences were observed in patients’ and OHCAs’ characteristics in period 1. Significant differences were instead highlighted in period 2: COVID-19 high-incidence provinces showed OHCA more frequently located at home, more frequently unwitnessed and of medical etiology. Moreover, the rate of bystander-CPR and of advanced resuscitation initiated were significantly lower in high-incidence provinces during period 2 as well as the resuscitation duration (S1 Table).

**Table 1:** Patients’ and OHCAs’ characteristics in the overall population and comparison between the different provinces.

*Variable*	*Overall*	*Pavia*	*Lodi*	*Cremona*	*Mantua*	*Varese*	*p*
	*n = 2785*	*n = 716*	*n = 250*	*n = 378*	*n = 445*	*n = 996*	
**Population**	2,415,491	540,376	227,412	355,908	406,919	884,876	
**Males, n (%)**	1659 (59.6)	415 (58)	155 (62)	232 (61.4)	268 (60.2)	588 (59.1)	<0.001
**Age, years [IQR]**	78 [65–86]	77 [64–85]	77 [63–73]	79 [67–87]	77 [63–85]	78 [65–86]	0.16
**EMS arrival time, mins [IQR]**	14.8 [11–18]	14 [11–18]	15 [12–20]	15 [11–19]	14 [11–17]	15 [12–19]	<0.01
**Etiology of arrest, n (%)**							0.1*
*Medical*	2408 (86.5)	646 (90.2)	201 (80.4)	342 (90.5)	350 (78.7)	869 (87.2)	0.27¶
*Trauma*	143 (5.1)	40 (5.6)	17 (6.8)	19 (5)	28 (6.3)	39 (3.9)	
*Drowning*	13 (0.5)	5 (0.7)	1 (0.4)	0 (0)	2 (0.4)	5 (0.5)	
*Overdose*	18 (0.6)	6 (0.8)	5 (2)	1 (0.3)	1 (0.2)	5 (0.5)	
*Electrocution*	0 (0)	0 (0)	0 (0)	0 (0)	0 (0)	0 (0)	
*Asphyxial (external causes)*	89 (3.2)	18 (2.5)	6 (2.4)	16 (3.6)	16 (4)	34 (3.4)	
*Unknown*	114 (4.1)	1 (0.1)	20 (8)	1 (0.3)	48 (10.8)	44 (4.4)	
**OHCA location, n (%)**							0.09
*Home*	2379 (85.4)	587 (82.0)	213 (85.2)	327 (86.5)	380 (85.4)	872 (87.6)	0.03¥
*Nursing residence*	113 (4.1)	40 (5.6)	12 (4.8)	16 (4.2)	20 (4.5)	25 (2.5)	
*Workplace*	25 (0.9)	9 (1.3)	2 (0.8)	5 (1.3)	5 (1.1)	4 (0.4)	
*Street*	206 (7.4)	61 (8.5)	19 (7.6)	26 (6.9)	36 (8.1)	64 (6.4)	
*Public building*	23 (0.8)	3 (0.4)	2 (0.8)	2 (0.5)	1 (0.2)	15 (1.5)	
*Sport*	1 (0.04)	1 (0.1)	0 (0)	0 (0)	0 (0)	0 (0)	
*School*	3 (0.1)	2 (0.3)	0 (0)	0 (0)	0 (0)	1 (0.1)	
*Other*	35 (1.3)	13 (1.8)	2 (0.8)	2 (0.5)	3 (0.7)	15 (1.5)	
**Witnessed status, n (%)**							<0.01*
*Unwitnessed*	1369 (49.2)	347 (48.5)	131 (52.4)	174 (46)	194 (43.6)	523 (52.5)	
*Bystander witnessed*	1052 (37.8)	287 (40.1)	84 (33.6)	166 (43.9)	162 (36.4)	353 (35.4)	
*Witnessed by EMS*	266 (9.6)	80 (11.2)	29 (11.6)	36 (9.5)	51 (11.5)	70 (7)	
*Unknown*	98 (3.5)	2 (0.3)	6 (2.4)	2 (0.5)	38 (8.5)	50 (5)	
**Resuscitation attempted by EMS, n (%)**							0.88*
*Yes*	1816 (65.2)	475 (66.3)	159 (63.6)	252 (66.7)	286 (64.3)	644 (64.7)	
*No*	965 (34.6)	241 (33.7)	91 (36.4)	126 (33.3)	156 (35.1)	351 (35.2)	
*Unknown*	4 (0.1)	0 (0)	0 (0)	0 (0)	3 (0.7)	1 (0.1)	
**Bystander CPR, n (%)** †							0.07*
*Yes*	604 (39.2)	169 (42.8)	61 (46.6)	82 (37.4)	94 (39.3)	198 (35.5)	
*No*	931 (60.4)	226 (57.2)	69 (52.7)	137 (62.6)	142 (59.4)	357 (64.1)	
*Unknown*	6 (0.4)	0 (0)	1 (0.8)	0 (0)	3 (1.3)	2 (0.4)	
**Dispatcher-assisted CPR, n (%)** †							0.36*
*Yes*	458 (29.7)	132 (33.4)	41 (31.3)	61 (27.9)	62 (25.9)	162 (29.1)	
*No*	1063 (69)	263 (66.6)	90 (68.7)	157 (71.7)	175 (73.2)	378 (67.9)	
*Unknown*	20 (1.3)	0 (0)	0 (0)	1 (0.5)	2 (0.8)	17 (3.1)	
**Presenting rhythm, n (%)** ‡							0.72*
*Shockable*	253 (13.9)	75 (15.8)	21 (13.2)	34 (13.5)	36 (12.6)	87 (13.5)	
*Not shockable*	1555 (85.6)	397 (83.6)	138 (86.8)	217 (86.1)	250 (87.4)	553 (85.9)	
*Unknown*	8 (0.4)	3 (0.6)	0 (0)	1 (0.4)	0 (0)	4 (0.6)	
**ACLS initiated, n (%)** ‡							<0.001*
*Yes*	909 (50.1)	218 (45.9)	85 (53.5)	166 (65.9)	177 (61.9)	263 (40.8)	
*No*	834 (45.9)	250 (52.6)	71 (44.7)	85 (33.7)	102 (35.7)	326 (50.6)	
*Unknown*	73 (4)	7 (1.5)	3 (1.9)	1 (0.4)	7 (2.4)	55 (8.5)	
**Resuscitation duration, mins [IQR]** ‡	22.1 (11.4–37)	23.2 (11.1–35.5)	25.9 (14.2–38.8)	25.8 (13.3–38.4)	22.7 (11–39.9)	19.2 (10.8–32.7)	<0.001*
**ROSC, n (%)** ‡							0.55*
*Yes*	229 (12.6)	61 (12.8)	26 (16.4)	33 (13.1)	36 (12.6)	73 (11.3)	
*No*	1579 (86.9)	414 (87.2)	133 (83.6)	214 (84.9)	249 (87.1)	569 (88.4)	
*Unknown*	8 (0.4)	0 (0)	0 (0)	5 (2)	1 (0.3)	2 (0.3)	

EMS: Emergency medical service; OHCA: Out-of-hospital cardiac arrest; CPR: Cardio-pulmonary resuscitation; ACLS: Advanced cardiac life support (i.e. endotracheal intubation, administration of drugs, mechanical CPR); ROSC: Return of spontaneous circulation.

* p-values are calculated excluding unknown cases

^¶^ p-value calculated for medical vs non-medical etiologies, excluding unknown cases

^¥^ p-value calculated for home vs non-home locations

^†^ Among those in whom resuscitation was attempted by EMS and excluding those witnessed by EMS

^**‡**^ Among those in whom resuscitation was attempted by EMS

**Table 2:** Comparison of the patients’ and OHCAs’ characteristics between the two periods.

*Variable*	*Period 1*	*Period 2*	*p*
	*n = 1408*	*n = 1377*	
**Males, n (%)**	857 (60.9)	802 (58.2)	0.32
**Age, years [IQR]**	78 (66–85)	78 (64–86)	0.6
**EMS arrival time, mins [IQR]**	14.5 (11–19)	15 (11–18)	0.8
**Etiology of arrest, n (%)**			0.98*
*Medical*	1187 (84.3)	1221 (88.7)	0.76¶
*Trauma*	69 (4.9)	74 (5.4)	
*Drowning*	7 (0.5)	6 (0.4)	
*Overdose*	8 (0.6)	10 (0.7)	
*Electrocution*	0 (0)	0 (0)	
*Asphyxial (external causes)*	43 (3.1)	46 (3.3)	
*Unknown*	94 (6.7)	20 (1.5)	
**OHCA location, n (%)**			0.06
*Home*	1230 (87.4)	1149 (83.4)	<0.01¥
*Nursing residence*	50 (3.6)	63 (4.6)	
*Workplace*	10 (0.7)	15 (1.1)	
*Street*	89 (6.3)	117 (8.5)	
*Public building*	10 (0.7)	13 (0.9)	
*Sport*	0 (0)	1 (0.1)	
*School*	0 (0)	3 (0.2)	
*Other*	19 (1.3)	16 (1.2)	
**Witnessed status, n (%)**			0.09*
*Unwitnessed*	688 (48.9)	681 (49.5)	
*Bystander witnessed*	507 (36)	545 (39.6)	
*Witnessed by EMS*	148 (10.5)	118 (8.6)	
*Unknown*	65 (4.6)	33 (2.4)	
**Resuscitation attempted by EMS, n (%)**			0.13*
*Yes*	893 (63.4)	923 (67)	
*No*	513 (36.4)	452 (32.8)	
*Unknown*	2 (0.1)	2 (0.1)	
**Bystander CPR, n (%)** †			0.14*
*Yes*	279 (37.4)	325 (40.8)	
*No*	466 (62.6)	465 (58.4)	
*Unknown*	0 (0)	6 (0.8)	
**Dispatcher-assisted CPR, n (%)** †			<0.001*
*Yes*	183 (24.6)	275 (34.5)	
*No*	554 (74.4)	509 (63.9)	
*Unknown*	8 (1.1)	12 (1.5)	
**Presenting rhythm, n (%)** ‡			0.55*
*Shockable*	120 (13.4)	133 (14.4)	
*Not shockable*	769 (86.1)	786 (85.2)	
*Unknown*	4 (0.4)	4 (0.4)	
**ACLS initiated, n (%)** ‡			0.001*
*Yes*	407 (45.6)	502 (54.4)	
*No*	436 (48.8)	398 (43.1)	
*Unknown*	50 (5.6)	23 (2.5)	
**Resuscitation duration, mins [IQR]** ‡	21.7 (10.8–37.2)	22.6 (12.1–36.7)	0.8*
**ROSC, n (%)** ‡			0.59*
*Yes*	109 (12.2)	120 (13)	
*No*	782 (87.6)	797 (86.3)	
*Unknown*	2 (0.2)	6 (0.7)	

EMS: Emergency medical service; OHCA: Out-of-hospital cardiac arrest; CPR: Cardio-pulmonary resuscitation; ACLS: Advanced cardiac life support (i.e. endotracheal intubation, administration of drugs, mechanical CPR); ROSC: Return of spontaneous circulation.

* p-values are calculated excluding unknown cases

^¶^ p-value calculated for medical vs non-medical etiologies, excluding unknown cases

^¥^ p-value calculated for home vs non-home locations

^†^ Among those in whom resuscitation was attempted by EMS and excluding those witnessed by EMS

^**‡**^ Among those in whom resuscitation was attempted by EMS

### COVID-19 and OHCA trend and correlation

**Fig 2:**
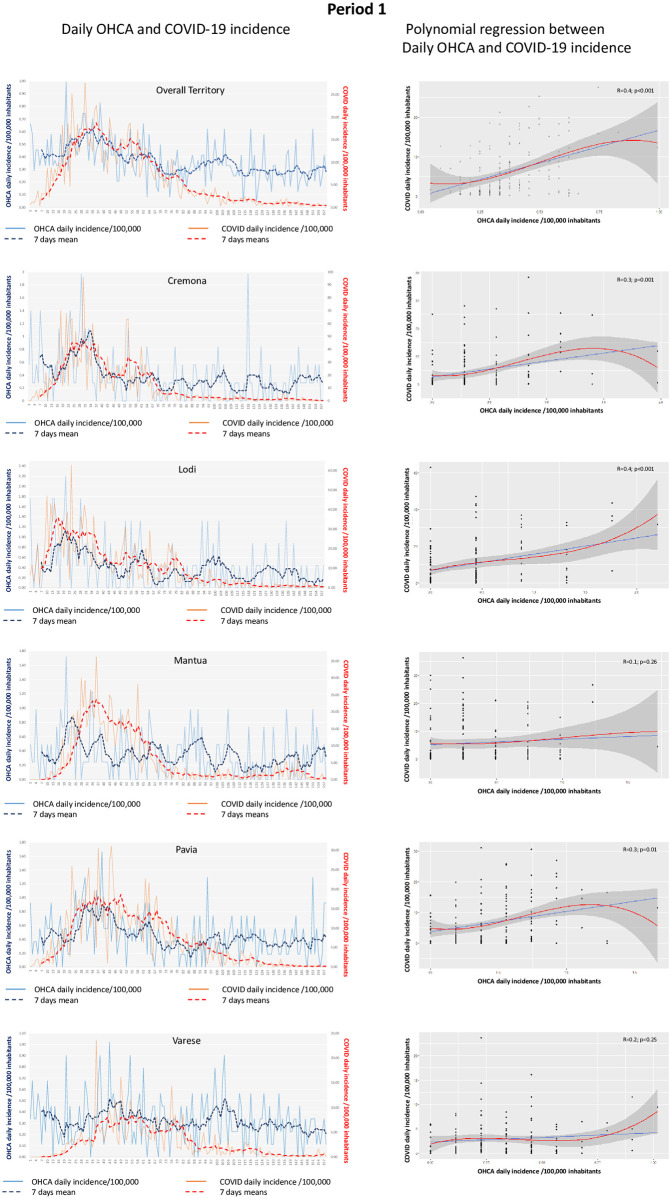
**Left side)** Trend of daily (blue continuous line) and 7-days (blue dotted line) OHCA incidence per 100,000 inhabitants and of daily (red continuous line) and 7-days (red dotted line) COVID-19 incidence per 100,000 inhabitants for the whole territory and each provinces separately in period 1. **Right side)** Relationship between COVID-19 daily incidence per 100,000 inhabitants (vertical axis) and OHCA daily incidence per 100,000 inhabitants (horizontal axis) in the whole territory and in each provinces separately in period 1. The blue line indicates the linear regression and the red line indicates the polynomial regression. The grey area indicates the 95%CI.

**Fig 3:**
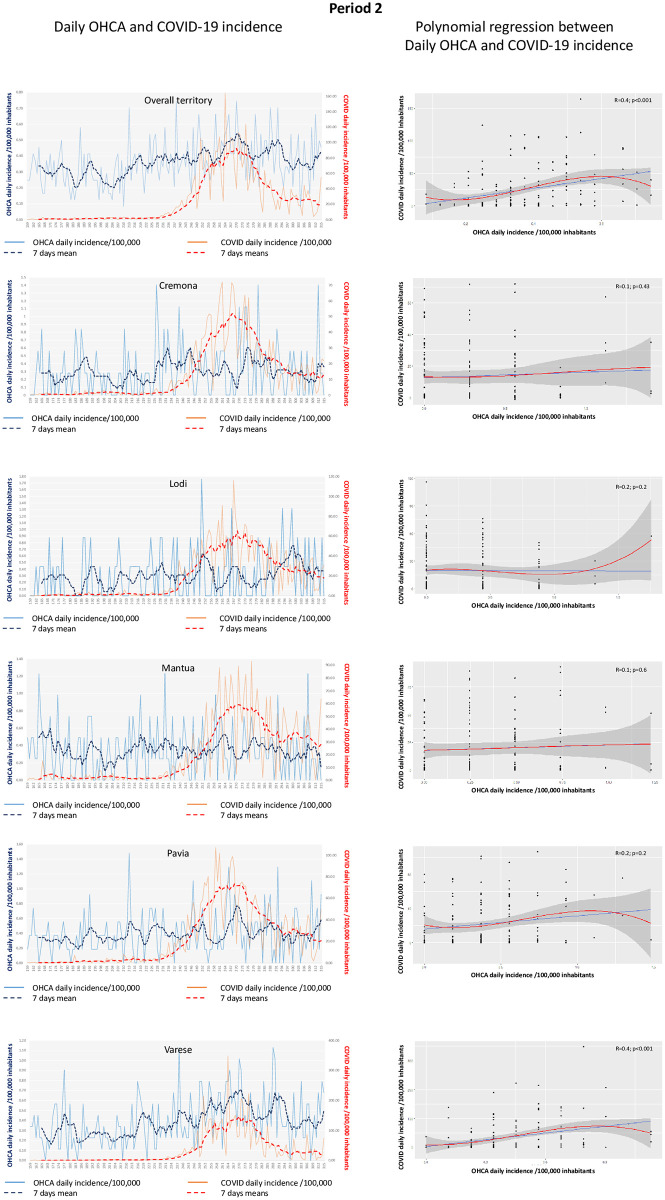
**Left side)** Trend of daily (blue continuous line) and 7-days (blue dotted line) OHCA incidence per 100,000 inhabitants and of daily (red continuous line) and 7-days (red dotted line) COVID-19 incidence per 100,000 inhabitants for the whole territory and each provinces separately in period 2. **Right side)** Relationship between COVID-19 daily incidence per 100,000 inhabitants (vertical axis) and OHCA daily incidence per 100,000 inhabitants (horizontal axis) in the whole territory and in each provinces separately in period 2. The blue line indicates the linear regression and the red line indicates the polynomial regression. The grey area indicates the 95%CI.

OHCA and COVID-19 daily and 7-days incidence per 100,000 inhabitants for the overall territory along the entire study period are presented in Fig 1B. A significant correlation between COVID-19 daily incidence and OHCA daily incidence was observed (R = 0.3, p<0.001 –Fig 1C). Considering the two periods separately, we found similar results as both in period 1 and in period 2: a significant correlation between the COVID-19 and the OHCA daily incidence was present in the entire territory (R = 0.4, p<0.001 for period 1 and 2) and only in the provinces at high COVID-19 incidence (period 1: Cremona R = 0.3, p = 0.001; Lodi R = 0.4, p<0.001; Pavia R = 0.3; p = 0.01; period 2: Varese R = 0.4, p<0.001). Similarly, in the two periods no significant correlation was found in the provinces at low COVID-19 incidence (period 1: Mantua R = 0.1, p = 0.26 and Varese R = 0.2, p = 0.25; period 2: Cremona R = 0.1, p = 0.43; Lodi R = 0.2, p = 0.2; Mantua R = 0.1, p = 0.6 and Pavia R = 0.2, p = 0.2) (Figs 2 and 3).

## Discussion

The present study is the first one providing data about the correlation between OHCA and COVID-19 incidence during both the first and the second pandemic wave. We highlight that a significant correlation between the daily incidence of COVID-19 and the daily incidence of OHCA was observed in the whole territory and only in those provinces whose cumulative incidence of COVID-19 has overcome those of the whole territory. This trend was confirmed both during the first and the second pandemic period, suggesting the importance of the impact of the burden of COVID-19 cases in a given area on the occurrence of OHCAs. Despite the correlation was not strong, we believe that our results highlight the importance of strictly monitoring the contagion trend, not only in the entire territory, but also in the different provinces. This may help to predict which territories will be more likely to experience an increase of OHCAs incidence and may also serve as a guide to re-consider the allocation of the pre-hospital resources in case of future pandemic waves.

The raising of OHCA incidence has been described during the first pandemic wave, compared to the previous year, in regions highly burdened by COVID-19 [1–3, 10, 20–22]. Such an increase was not observed in those regions with a low-incidence of COVID-19 cases [23–27], suggesting a role of the pandemic burden probably due to both the direct and the indirect effects of the outbreak. Two of the main issues possibly related to that increase were the fear of in-hospital infection and the “surprise effect” of the pandemic on the healthcare systems, typical of the first pandemic wave [10]. It was unknown if information campaigns, adopted to increase citizens’ awareness to call EMS in case of effected need, and re-organization of EMS and healthcare systems [28, 29] may have prevented a similar increase during the second wave, avoiding the correlation between COVID-19 and OHCA incidence. Our study highlights that also in one of the first Region early affected by the pandemic, where the first case of COVID-19 in Europe was documented, and where multiple actions were adopted to mitigate the pandemic effect, an increase in OHCAs with a significant correlation between COVID-19 and OHCA incidence was observed also during the second pandemic wave, but only in high COVID-19 incidence provinces.

An interesting element worth to be deeply discussed and that could have potential practical implications in case of subsequent peaks, is that a major role seems to be played by the incidence of COVID-19 cases in the different provinces of our territory. Our study is indeed the first evaluating different areas, but encompassed in a homogenous region, served by the same EMS system, avoiding bias due to different organization of the pre-hospital ambulance system and hospital resources. We highlighted that during both the pandemic waves, the trend of OHCAs was significantly correlated to the trend of COVID-19 only in those provinces deeply affected by the pandemic, with an incidence of COVID-19 cases overcoming those of the whole territory analysed. Only few previous studies carried out so far on this topic have included an analysis on the different areas. A study performed in the United States [21] showed how the communities with a high COVID-19 mortality experienced a higher increase of OHCAs as compared to 2019 in contrast with the communities with a lower COVID-19 mortality. A correlation between COVID-19 cases and OHCA incidence has also been described by a previous study of our group carried out in the south part of the Lombardy Region [10] and by a study from London [22], both focused on the first 60 days of the pandemic. On the other hand, another study from Switzerland [30] didn’t highlighted an excess of OHCA in 2020 as compared to 2019. This was found also in those Cantons with a high incidence of COVID-19; however, albeit high for Switzerland, the incidence of COVID-19 in the Swiss Cantons was less than half of our high-incidence provinces, resembling the scene of our low-incidence provinces. Therefore, the lack of OHCA incidence increase in Switzerland could be attributed to the fact that that territories was not burdened by COVID-19 enough to cause an increase of OHCAs cases. All the studies discussed before were focused on the first pandemic wave, therefore it could be speculated that other factors could have affected the increasing of OHCA besides COVID-19 incidence, like as EMS organization, population’s characteristics and different beds and ICU hospital availability. Our study, covering both the first and the second pandemic wave, overcome this limitation highlighting that in provinces in the same region, with the same EMS organization and the same healthcare system, the increase of OHCA and the correlation between COVID-19 and OHCA incidence are evident only if the COVID-19 incidence is particularly higher.

The reasons for this evidence have to be sought in the direct and indirect effect of the pandemic [31]. In the areas deeply affected by COVID-19 outbreak, it is reasonable that there was an increase in OHCAs due to both cardiovascular and respiratory causes directly liked to SARS-CoV-2 infection [32, 33], caused by the increase in absolute number of the COVID-19 cases and, therefore, of the most critical clinical pictures. However, in these areas, there was also probably an increase of OHCA due to at-home deterioration of time-dependent diseases due to the fear of patients to in-hospital contagion, which prevented them to activate the EMS [10, 31]. This was probably more evident in these areas, compared to the low-incidence ones, as the population knew the load of people hospitalized for COVID-19 in the hospital of their province resulting in an increased fear of in-hospital contagion. Based on the above, we believe that our results could have great relevance. Considering indeed the risk of subsequent pandemic waves, mostly due to the spread of different variants of the virus [34, 35], and the future possible pandemic due to other viruses, the governments and healthcare politics will have to strictly monitor the contagion trend in order to be able to identify the territories higher at risk to be hit by a surge of OHCAs.

Regarding the patients’ and OHCAs’ characteristics, it is interesting to point out how the percentage of OHCAs occurred at home was higher during period 1 than in period 2. This observation can be the result of the stricter lockdown in period 1 than in period 2. Moreover, the dispatcher assisted-CPR was less provided in period 1 than in period 2 probably to prevent bystanders from contagion in the absence of defined knowledge of the new virus and of indication on how to safely perform CPR. Then, at the time of the second pandemic wave, some specific recommendations and guidelines were issued by the International Liaison Committee on Resuscitation (ILCOR) and other scientific societies [36–38], reassuring the dispatchers about the safety and feasibility of telephone-CPR also during a pandemic. Similarly, also advanced resuscitation was attempted more in period 2 than in period 1, probably favoured by the increased availability of hospital resources during the second pandemic wave and by the satisfying results of advanced resuscitation experienced during the first phase [39]. These data can help to understand how the actions implemented to fight against the spread of pandemic, the different organizations during the two waves and the specific guidelines adopted after the first phase of COVID-19 pandemic may have played a role by modifying patients’ and OHCAs’ characteristics.

Our study has some limitations. The main limitation of our study is that the COVID-19 incidence was calculated on the confirmed cases announced by the National Department of Civil Defence. The number of cases is therefore strictly dependent on the number of Real-Time PCR test performed, which has considerably increased in the second compared to the first wave. However, this change in RT-PCR test performed has been homogenous in our territory as it is the same Region with the same healthcare system. Moreover, by considering the COVID-19 incidence in the different provinces compared to the whole territory separately for the first and second pandemic wave, we should have overcome this limitation. Probably, the number of deaths attributable to COVID-19 would be a data less exposed to change between the two waves, but, unfortunately, it is not available for every single province in our country as well as the number of patients admitted in the intensive care units. The second limitation is that, in this manuscript, we were not able to assess the number of patients with confirmed or suspected COVID-19 and also the outcome at hospital admission or discharge. The third limitation is that we cannot use 2019 data as comparison because the province of Varese was included in the LombardiaCARe in January 2020, therefore we have not historical data for this territory. However, we believe that the comparison of the provinces with the whole territory separately during the first and second pandemic wave can mitigate this limitation. Moreover, the comparison with 2019 was not an aim of the present study. Furthermore, the lacking of a control historical population do not allow to exclude with certainty that some of the differences observed in the two periods, and between high and low COVID-incidence provinces, are due to differences already present before the pandemic. However, considering that our population is quite homogeneous as all the territories pertains to the Lombardia Region, the data present in literature regarding the changes in OHCAs’ characteristics during the pandemic and, moreover, that the Emergency Medical System is the same for all the territories (Agenzia Regionale Emergenza Urgenza—Lombardia), we believe that our results are more likely representing differences bounded to the pandemic rather than the different provinces.

## Conclusions

A correlation between the daily incidence of COVID-19 and OHCAs was observed only in the areas with a cumulative incidence of COVID-19 exceeding those of the whole territory. This was highlighted both during the first and the second pandemic wave. Our results suggest that strictly monitoring the contagion trend comparing every province to the whole territory may help to early identify those territories at higher risk of an increase of OHCA incidence. Our results may also serve as a guide to re-consider the allocation of the pre-hospital resources and to tailor information campaigns in the future pandemic waves.

## Supporting information

S1 Data (XLSX)

S1 Table Comparison of patients’ and OHCAs’ characteristics in the two periods according to the level of COVID-19 incidence (provinces with high-COVID incidence vs provinces with low-COVID incidence). (DOCX)
