# Supplementary material for: Relationship between out-of-hospital cardiac arrests and COVID-19 during the first and second pandemic wave. The importance of monitoring COVID-19 incidence
Source: PLoS One. 2021 Nov 19;16(11):e0260275. doi: 10.1371/journal.pone.0260275 (PMC8604324; doi:10.1371/journal.pone.0260275)
Supplement: S1 Table — (DOCX) [file pone.0260275.s002.docx]

**S1 Table.** Comparison of patients’ and OHCAs’ characteristics in the two periods according to the level of COVID-19 incidence (Provinces with high-COVID incidence vs Provinces with low-COVID incidence)

|  |  | **Period 1**  **n=1408** | |  |  | **Period 2**  **n=1377** | |  |
| --- | --- | --- | --- | --- | --- | --- | --- | --- |
| ***Variable*** |  | ***High COVID incidence***  ***n=734*** | ***Low COVD Incidence***  ***n=674*** | ***p value*** |  | ***High COVID incidence***  ***n=547*** | ***Low COVD Incidence***  ***n=830*** | ***p value*** |
| **Provinces** |  | Cremona, Lodi, Pavia | Mantua, Varese |  |  | Varese | Cremona, Lodi, Mantua, Pavia |  |
| **Population** |  | 1,123,696 | 1,291,795 |  |  | 884,876 | 1,530,615 |  |
| **Males, n (%)** |  | 446 (60.8) | 411 (61) | 0.69 |  | 321 (58.7) | 481 (58) | 0.34 |
| **Age, years [IQR]** |  | 77 (65.3-84) | 78 (66-86) | 0.05 |  | 79 (66.8-86) | 77 (62-86) | 0.2 |
| **EMS arrival time, mins [IQR]** |  | 14 (11-18.8) | 15.8 (11.5-18.9) | 0.07 |  | 15.3 (11.9-18.7) | 14 (11-18) | 0.2 |
| **Etiology of arrest, n (%)** |  |  |  | 0.95* |  |  |  | 0.03* |
| *Medical* |  | 647 (88.1) | 540 (80.1) | 0.71¶ |  | 496 (90.7) | 725 (87.3) | 0.06¶ |
| *Trauma* |  | 36 (4.9) | 33 (4.9) |  |  | 17 (3.1) | 57 (69) |  |
| *Drowning* |  | 3 (0.4) | 4 (0.6) |  |  | 3 (0.5) | 3 (0.4) |  |
| *Overdose* |  | 5 (0.7) | 3 (0.4) |  |  | 3 (0.5) | 7 (0.8) |  |
| *Electrocution* |  | 0 (0) | 0 (0) |  |  | 0 (0) | 0 (0) |  |
| *Asphyxial (external causes)* |  | 23 (3.1) | 20 (3) |  |  | 21 (3.8) | 25 (3) |  |
| *Unknown* |  | 20 (2.7) | 74 (11) |  |  | 7 (1.3) | 13 (1.6) |  |
| **OHCA location, n (%)** |  |  |  | 0.18 |  |  |  | <0.01 |
| *Home* |  | 633 (86.2) | 597 (88.6) | 0.19¥ |  | 476 (87) | 673 (81.1) | <0.01 |
| *Nursing residence* |  | 33 (4.5) | 17 (2.5) |  |  | 15 (2.7) | 48 (5.8) |  |
| *Workplace* |  | 7 (1) | 3 (0.4) |  |  | 2 (0.4) | 13 (1.6) |  |
| *Street* |  | 47 (6.4) | 42 (6.2) |  |  | 36 (6.6) | 81 (9.8) |  |
| *Public building* |  | 3 (0.4) | 7 (1) |  |  | 9 (1.6) | 4 (0.5) |  |
| *Sport* |  | 0 (0) | 0 (0) |  |  | 0 (0) | 1 (0.1) |  |
| *School* |  | 0 (0) | 0 (0) |  |  | 1 (0.2) | 2 (0.2) |  |
| *Other* |  | 11 (1.5) | 8 (1.2) |  |  | 8 (1.5) | 8 (1) |  |
| **Witnessed status, n (%)** |  |  |  | 0.19* |  |  |  | <0.01* |
| *Unwitnessed* |  | 357 (48.6) | 331 (49.1) |  |  | 293 (53.6) | 388 (46.7) |  |
| *Bystander witnessed* |  | 280 (38.1) | 227 (33.7) |  |  | 199 (36.4) | 346 (41.7) |  |
| *Witnessed by EMS* |  | 88 (12) | 60 (8.9) |  |  | 34 (6.2) | 84 (10.1) |  |
| *Unknown* |  | 9 (1.2) | 56 (8.3) |  |  | 12 (1.4) | 21 (3.8) |  |
| **Resuscitation attempted by EMS, n (%)** |  |  |  | 0.93* |  |  |  | 0.14 |
| *Yes* |  | 467 (63.6) | 426 (63.2) |  |  | 354 (64.7) | 569 (68.6) |  |
| *No* |  | 267 (36.4) | 246 (36.5) |  |  | 192 (35.1) | 260 (31.3) |  |
| *Unknown* |  | 0 (0) | 2 (0.3) |  |  | 1 (0.2) | 1 (0.1) |  |
| **Bystander CPR, n (%)** † |  |  |  | 0.055 |  |  |  | <0.01* |
| *Yes* |  | 155 (40.8) | 124 (34) |  |  | 107 (34.9) | 218 (44.6) |  |
| *No* |  | 225 (59.2) | 241 (66) |  |  | 198 (64.5) | 267 (54.6) |  |
| *Unknown* |  | 0 (0) | 0 (0) |  |  | 2 (0.7) | 4 (0.8) |  |
| **Dispatcher-assisted CPR, n (%)** † |  |  |  | 0.53* |  |  |  | 0.12 |
| *Yes* |  | 98 (25.8) | 85 (23.3) |  |  | 94 (30.6) | 181 (37) |  |
| *No* |  | 282 (74.2) | 272 (74.5) |  |  | 203 (66.1) | 306 (62.6) |  |
| *Unknown* |  | 0 (0) | 8 (2.2) |  |  | 10 (3.3) | 2 (0.4) |  |
| **Presenting rhythm, n (%) ‡** |  |  |  | 0.22* |  |  |  | 0.71* |
| *Shockable* |  | 69 (14.8) | 51 (12) |  |  | 49 (13.8) | 84 (14.8) |  |
| *Not shockable* |  | 396 (84.8) | 373 (87.6) |  |  | 303 (85.6) | 483 (84.9) |  |
| *Unknown* |  | 2 (0.4) | 2 (0.5) |  |  | 2 (0.6) | 2 (0.4) |  |
| **ACLS initiated, n (%) ‡** |  |  |  | 0.54* |  |  |  | <0.001* |
| *Yes* |  | 226 (48.4) | 181 (42.5) |  |  | 156 (44.1) | 346 (60.8) |  |
| *No* |  | 233 (49.9) | 203 (47.7) |  |  | 179 (50.6) | 219 (38.5) |  |
| *Unknown* |  | 8 (1.7) | 42 (9.9) |  |  | 19 (5.4) | 4 (0.7) |  |
| **Resuscitation duration, mins [IQR] ‡** |  | 23.2 (10.5-38.9) | 20.5 (11-36) | 0.3* |  | 19.7 (10.8-33.2) | 24.2 (13-38.3) | 0.02 |
| **ROSC, n (%) ‡** |  |  |  | 0.3* |  |  |  | 0.22 |
| *Yes* |  | 62 (13.3) | 47 (11) |  |  | 40 (11.3) | 80 (14.1) |  |
| *No* |  | 403 (86.3) | 379 (89) |  |  | 312 (88.1) | 485 (85.2) |  |
| *Unknown* |  | 2 (0.4) | 0 (0) |  |  | 2 (0.6) | 4 (0.7) |  |
